# Association of drinking water and environmental sanitation with diarrhea among under-five children: Evidence from Kersa demographic and health surveillance site, eastern Ethiopia

**DOI:** 10.3389/fpubh.2022.962108

**Published:** 2022-11-14

**Authors:** Gutema Mulatu, Galana Mamo Ayana, Haileyesus Girma, Yohannis Mulugeta, Gamachis Daraje, Abraham Geremew, Merga Dheresa

**Affiliations:** ^1^Department of Environmental Health, College of Health and Medical Science, Haramaya University, Dire Dawa, Ethiopia; ^2^Department of Epidemiology and Biostatistics, School of Public Health, College of Health and Medical Science, Haramaya University, Dire Dawa, Ethiopia; ^3^Department of Statistics, College of Computing and Informatics, Haramaya University, Dire Dawa, Ethiopia; ^4^School of Nursing and Midwifery, College of Health and Medical Science, Haramaya University, Dire Dawa, Ethiopia

**Keywords:** Kersa, diarrhea, predictors, sanitation, supply, regression

## Abstract

**Background:**

Diarrhea remains one of the leading causes of mortality and morbidity, despite the global progression of eradicating the burden of diarrhea-related morbidity and mortality in the past two decades. In Sub-Saharan African (SSA) countries, there is inadequate supply and sanitation of safe water. However, there is a lack of literature that estimates the impact of drinking water and sanitation service on childhood diarrhea in Kersa Demographic and Health Surveillance. Therefore, the current study aimed to assess the prevalence and effect of water supply and environmental sanitation on diarrhea among under-five children from 2017 to 2021 in Kersa Demographic and Health Surveillance, Eastern Ethiopia.

**Method:**

A prospective cohort study design was implemented among 6,261 children from the Kersa Health Demographic Surveillance System (HDSS), Eastern Ethiopia, from 1 January 2016 to 31 December 2021. STATA statistical software was used to extract data from the datasets. The binary logistic regression was used to identify the impact of water supply and environmental sanitation on diarrhea by controlling important confounders. The adjusted odds ratio (AOR) with a 95% confidence interval measures this association.

**Result:**

The current study showed that among 6,261 under-five children, 41.75% of them had developed active diarrhea during the follow-up time. The final model depicted that having media exposure of 22% [AOR - 0.78 CI: (0.61, 0.98)], a protected tube well source of drinking water of 50% [AOR - 1.50, CI: (1.32, 1.71)], unprotected tube well source of drinking water of 66% [AOR - 1.66 CI: (1.27, 2.18)], having toilet facility of 13% [AOR - 0.87 CI: (0.78, 0.97)], and accessibility of source of water [AOR - 1.17 CI: (1.05, 1.30)] showed a significant association with diarrhea among under-five children.

**Conclusion:**

The prevalence of diarrhea is found to be high in the Kersa District. The main predictors of diarrhea under five were a lack of latrines, an unimproved source of drinking water, and a distance from access to drinking water. The study setting should focus on increasing the adequacy of safe drinking water and sanitation.

## Introduction

Despite a global effort to reduce diarrheal disease, childhood diarrhea remains one of the leading causes of mortality and morbidity and accounts for one in nine deaths globally, particularly in developing countries (1). In 2019, diarrhea among under-five children was one of the causes of about 5.2 million deaths, which could have been prevented with access to simple and affordable interventions, including safe drinking water and sanitation services (1). In 2016, about 1.6 million deaths and 104.6 million global disability-adjusted life years (DALYs) were from Water, Sanitation, and Hygiene (WASH)-attributable disease burden (2).

Countries in Sub-Saharan Africa were the most impacted due to inadequate water and sanitation services; for instance, nearly 53% of all deaths and 60% of all DALYs occur in this region (3, 4). Moreover, the burden of inadequate WASH services is severe in children under 5 years, with nearly 297,000 deaths due to diarrhea (i.e., 5.3% of all deaths in this age group) (2). According to the World Health Organization (WHO), important measures for combating such public health issues include improved drinking water supply, sanitation facilities, and proper personal hygiene practices (3, 5).

In Ethiopia, diarrhea is the second leading cause of under-five children's death, with an estimated annual loss of more than 70,000 children (i.e., accounting for 23% of all under-five deaths) due to diarrheal diseases (6). An estimated 85–90% of diarrheal diseases are caused by inadequate sanitation, a lack of safe drinking water, and poor personal hygiene (7). Although the Ethiopian government and non-governmental organizations have devoted several projects and interventions to address these issues, the coverage and utilization are still low. For example, <50% of the population has basic drinking water services, and about 5% is dependent on surface water as a source of drinking water supply (8). The coverage of sanitation services is also minimal in the country. Only 9% have basic service, and about 17% of the population practices open defecation. There is no difference in the hygiene service coverage as the country is behind the target for achieving the 2030 Sustainable Development Goals (SDGs) with only 8% of the total population having basic hygiene services (8). Several scientific findings indicate that safe and adequate drinking water supply, environmental sanitation, and proper personal hygiene practice could significantly reduce the burden of childhood diarrheal disease (9–12).

Previous studies conducted in different regions of Ethiopia showed a high prevalence of childhood diarrhea, particularly associated with a poor water supply and sanitation services. For example, a meta-analysis study conducted in 31 Ethiopian districts showed that the prevalence of diarrhea among under-five children was 22% (95% CI: 19, 25%). The authors concluded that one reason for the high prevalence of diarrhea was a lack of latrine availability, with an odds ratio of two (12). Another meta-analysis study conducted in three sub-Saharan African countries, namely Kenya, Ethiopia, and Somalia, from 2012 to 2017 indicated that the average prevalence of diarrhea disease among under-five children was 27% (13). In addition, a cross-sectional study conducted in the Haramaya demographic and health surveillance site, in Eastern Ethiopia, revealed that the prevalence of diarrhea among under-five children was 24.8% (95% CI: 22.3–27.6). They highlighted that improvement of WASH service levels is needed as the prevalence of childhood diarrhea was lower in the higher service levels compared to the lower service levels (10). Moreover, a study in Northwest Ethiopia found that the great majority of the prevalence of diarrhea among under-five children is linked to households with contaminated drinking water, mainly by fecal contamination (14).

However, studies that determine the prevalence of diarrhea among under-five children from a pooled cross-sectional study with a wider time duration are missing, especially in the surveillance site. Therefore, this study is intended to analyze the prevalence and water supply and environmental sanitation predictors of diarrhea among under-five children from 1 January 2016 to 31 December 2021 in Kersa demographic and health surveillance site, Eastern Ethiopia.

## Materials and methods

### Study setting, period, and design

The study was conducted among under-five children who were under the follow-up system of the Kersa Health Demographic Surveillance System (HDSS) field site in Eastern Ethiopia from 1 January 2016 to 31 December 2021. The Kersa HDSS site is a full member of the International Network of Demographic Evaluation of Populations and Their Health (INDEPTH). The Kersa HDSS was recognized in September 2007 in the Eastern Hararghe Zone, Kersa district, and then the enumeration area of the system was expanded to the Harari region, Hara town in 2012. The enumeration area of Kersa HDSS contains 36 kebeles (the lowest administrative units) (15). Kersa HDSS follows an open dynamic cohort study design that follows people living within a specific geographical boundary.

### Population and sample

The source population of the study was all under-five children residing around Eastern Ethiopia. The study population included 6,261 under-five children in the Kersa HDSS, and children whose outcome variable was unknown were excluded.

### Kersa HDSS data collection system

Kersa HDSS has a well-trained common data collector who collects data regularly through face-to-face interviews using a tablet computer with the Open Data Kit (ODK) data collection application. Supervisors were assigned to supervise data collectors in the field. Field supervisors checked the quality of data before it was sent to the server. Supervisor closely follow data collectors and they communicate with the data collector for the correction if a quality problem is found. The collected data was temporarily stored on the ODK aggregate. The data manager approved the quality of the data and migrated the data from temporary storage to the final storage area of the Openhds database system.

### Database and data extraction system

Primarily, Kersa HDSS follows an open dynamic cohort study design that follows people living within a specific geographical boundary. For the current study analysis, researchers extracted a single time point of 7 years (1 January 2016–31 December 2021) data from the Kersa HDSS database system.

### Variables and measurements

The outcome variable for this study was the presence of diarrhea. The outcome variable was dichotomized and coded as 1 if the children experienced diarrhea and 0 for those who did not develop diarrhea. The extracted independent variables were children's age, mothers' age, mothers' occupation, mothers' educational status, household wealth quantiles, type of treatment for diarrhea, media exposure, source of water in households, the distance of water source, toilet facility, type of toilet facility, family size, and a number of under-five children.

#### Childhood diarrhea

Childhood diarrhea is defined as children under five experiencing three or more loose or watery stools in 1 day period that occurred at least once in the past 2 weeks period before the survey, as reported by the mothers/caretakers of the child.

#### Prevalence of diarrhea

The total number of diarrheal cases obtained among the study group at the specified period of time divided by the total number of under-five children in the study.

#### Improved water sources

Household connections, public standpipes, protected dug wells, and protected springs were among the improved water sources. Unprotected dug wells and unprotected springs are examples of “unimproved” water sources. A source that has been “improved” is more likely to offer “safe” water.

Sanitation of the environment includes the type of water source, distance to the water source, amount of daily water consumption, availability of latrine, refuse disposal, etc.

Improved latrines include flush toilets, piped sewer systems, septic tanks, vented improved pit latrines (VIP), and pit latrines with slabs.

#### Contact with other people

It includes toilets with flush or pours/flush connections to sewers or septic tanks, as well as composting toilets.

#### Unimproved toilet

A flush/pour flush to elsewhere, a pit latrine without slab or bucket, a hanging toilet or hanging latrine, no facilities, and a bush or a field.

#### Basic water service

Drinking water from a better source if the roundtrip collection time, including queue, is <30 min (16).

#### Limited water service

Drinking water from a better source with a collection duration of more than 30 min for a roundtrip, including queue (4).

#### Unimproved water source

Drinking water from an unprotected dug well or unprotected spring (4).

#### Safely managed sanitation

Using a better sanitation facility that is not shared with other homes and where excreta is safely disposed of on-site or transported and treated off-site (4).

#### Improved sanitation

Facilities must not be shared and must provide sanitary separation of human excreta from protected latrines such as VIP latrines or latrines with slabs (16).

Pit latrines without a slab or platform, hanging latrines, and bucket latrines were the common category of unimproved facilities.

#### Basic sanitation service

Use of improved facilities that are not shared with other households (4).

#### Limited sanitation

Shared use of modernized facilities by two or more households (4).

#### Open defecation

It is known as the disposal of human excrement in fields, forests, shrubs, open bodies of water, beaches, or other open locations, or alongside solid garbage (16).

### Ethical clearance

The Institutional Health Research Ethical Review Committee (IHRERC) of Haramaya University, College of Health and Medical Sciences provided ethical clearance to the Kersa Health Demographic Surveillance. All the procedures were in accordance with the principles of the Helsinki declaration.

### Data management and processing

Data extraction from Kersa HDSS datasets for useful dependent and independent variables, data cleaning, coding, recoding, and further statistical analysis was conducted using STATA version 14 statistical software.

### Statistical analysis

Descriptive statistics were conducted using frequency and percentages for categorical data, and mean values with standard deviation were used to summarize continuous data. The overall prevalence of diarrhea during the study period was calculated using proportion. A bi-variable binary logistic regression model was fitted for each explanatory variable, and a variable with a *p* < 0.2 was a candidate for multivariable. Finally, a multivariable binary logistic regression model was fitted to identify significant predictors of diarrhea. The variables with a *p*-value of 0.05 were identified as statistically significant predictors of diarrhea. An adjusted odds ratio with a 95% CI was used to measure the strength of the associations. The goodness of fit of the model was checked using a Hosmer and Lemeshow (HL) statistical test.

## Results

### Socio-demographic characteristics of children and mothers

A total of 6,261 children were included in the final analysis. Among the study participants, 3,278 (52.4%) were boys. A three-fourth (75.1%) of children's mothers had no formal education. Moreover, more than 91% (91.4%) of the mother's occupation was a farmer. The average household member of the study participants was 6.25807 with an SD of 2.073121. The average age of under-five children and mothers included in the final analysis was 2.55 with an SD of 1.36 and 29.48 with an SD of 7.06, respectively (Table 1).

**Table 1:** Socio-demographic characteristics of under-five children in Kersa Health Demographic Surveillance System (HDSS) from 2016 to 2021.

**Variables**	**Frequency (%)**
**Sex**
Male	3,278 (52.36)
Female	2,985 (47.65)
**Mother occupation**
House wife	43 (0.70)
Paid employers	219 (3.56)
Farmer	5,629 (91.44)
Merchant	90 (1.46)
Daily laborer	126 (2.05)
Other	49 (0.8)
**Educational status**
Unable to read and write	4,690 (75.05)
Read only	47 (0.75)
Can read and write	50 (0.80)
Literate	1,462 (23.40)
**Family size**
1–4	1,354 (21.74)
≥5	4,873 (78.26)
**Number of under 5 children**	
One	3,102 (49.68)
≥2	3,142 (50.32)
**Media exposure**
No	5,655 (91.86)
Yes	501 (8.14)
**Wealth quantiles**
First quintile	2,196 (35.67)
Second quintile	1,933 (31.40)
Third quintile	2,027 (32.93)

Other: Student, retired.

### Prevalence of diarrhea

Among 6,261 under-five children, 41.75% of them had developed active diarrhea during the follow-up time (Table 2).

**Table 2:** Water sanitation and treatment-related characteristics of under-five children in Kersa HDSS from 2016 to 2021.

**Variables**	**Frequency (%)**
**Child get treatment**
Yes	1,591 (60.68)
No	1,031 (39.32)
**Type of treatment**
Injection	945 (24.75)
Tablet	1,382 (36.21)
Herbal medicine	185 (4.85)
Homemade fluid	320 (8.38)
ORS	780 (20.43)
IV-fluid	205 (5.37)
**Child had any symptoms of malnutrition**
Yes	1,017 (26.7)
No	2,791 (73.54)
**Malnutrition symptoms type**
Wasting	783 (76.99)
Swelling	234 (23.01)
**Toilet facility**
Yes	3,130 (50.25)
No	3,099 (49.75)
**Type of toilet facility**	
Ventilated improved Pit latrine	18 (0.29)
Pit latrine with cover	3,112 (49.96)
**Source of drinking water**
Piped water	2,920 (46.64)
Protected tube well	1,708 (27.28)
Unprotected tube well	249 (3.98)
Protected spring	511 (8.16)
Unprotected spring	833 (13.30)
Other	40 (0.64)

Other: Surface water, river, and pond.

### Water sanitation, hygiene, and treatment-related characteristics

Among 2,614 children who developed diarrhea, three-fifths (60.7%) of them received treatment. Of those who received treatment, 945 (24.8%) of them were treated with injection and 1,382 (36.2%), 185 (4.9%), 320 (8.4%), 780 (20.43%), and 205 (5.4%) were treated with tablets, herbal medicine, homemade fluid, and ORS (IV-fluid), respectively. Moreover, among the study participants, 53.0% of the sources of water for the household exist within an accessible distance. A total of 46.9% of the main sources of water of households were piped water sources. Among the study participants, nearly half (49.6%) of them have no latrine facility (Table 2).

### Predictors of diarrhea

A mother's educational level, wealth index, the main source of drinking water, having media exposure, having toilet facility, and accessibility to the source of water showed significant associations with diarrhea among under-five children. However, the final model multivariable logistic regression revealed that having media exposure, the main source of drinking water, having a toilet facility, and accessibility of the source of water are all significant predictors of diarrhea among under-five children.

The final model showed that having media exposure reduces the odds of diarrhea among under-five children by 22% [AOR 0.78 CI: (0.61, 0.98)] when compared to their counterparts. The odds of developing diarrhea was 50% [AOR 1.50, CI: (1.33, 171)] more likely among respondents whose main source of drinking water was a protected tube well when compared to those who utilized piped water. Furthermore, the odds of developing diarrhea were 66% [AOR 1.66 CI: (1.27, 2.18)] more likely among the study participants whose main source of drinking water was an unprotected tube well when compared to those whose main source of drinking water was piped water. The present study also revealed that the availability of toilet facilities in the household reduces diarrhea among under-five children by 13% [AOR: 0.87 CI: (0.78, 0.97)] when compared to their counterparts. Besides, the accessibility of the source of water showed a significant association with diarrhea among under-five children. The odds of developing diarrhea among under-five children was 17% [AOR: 1.17 CI: (1.05, 1.30)] more likely among respondents who accessed the source of water from a long distance when compared to individuals who can easily access water (Table 3).

**Table 3:** Predictors associated with diarrhea among under five children in Kersa HDSS from 2015 to 2021.

**Variables**	**Diarrhea**		**COR (95% CI)**	**AOR (95% CI)**
	**Yes**	**No**		
**Mother educational status**
Literate	551	912	1	1
Read only	16	31	0.85 (0.46, 1.57)	0.76 (0.41, 1.42)
Can read and write	25	28	1.47 (0.85, 2.56)	1.50 (0.84, 2.66)
Unable to read and write	2,020	2,665	1.25 (1.11, 1.41)	1.09 (0.96, 1.25)
**Media exposure**
Yes	159	342	0.62 (0.51, 0.75)	0.78 (0.61, 0.98)^*^
No	2,415	3,240	1	1
**Wealth index**
First quintile	975	1,221	1	1
Second quintile	831	1,102	0.94 (0.83, 1.06)	0.98 (0.87, 1.13)
Third quintile	768	1,259	0.76 (0.67, 0.86)	0.90 (0.78, 1.04)
**Source of drinking water**
Piped water	1,103	1,817	1	1
Protected tube well	837	871	1.58 (1.40, 1.78)	1.50 (1.32, 1.71)^**^
Unprotected tube well	134	115	1.91 (1.47, 2.48)	1.66 (1.27, 2.18)^**^
Protected spring	171	340	0.82 (0.67, 1.01)	1.02 (0.88, 1.17)
Unprotected spring	356	477	1.22 (1.05, 1.43)	0.56 (0.11, 2.80)
Other	13	27	0.79 (0.40, 1.54)	0.84 (0.52, 2.84)
**Toilet facility**
Yes	1,236	1,894	0.82 (0.75, 0.91)	0.87 (0.78, 0.97)^*^
No	1,367	1,732	1	1
**Distance from source water**
Accessible	1,414	1,905	1	1
Not accessible	1,200	1,742	1.07 (0.97, 1.96)	1.17 (1.05, 1.30)^**^

Other: Surface water, river, and pond.

* P < 0.05,

** P < 0.01, and p < 0.001.

## Discussion

The leading cause of morbidity in Ethiopia and other developing countries is diarrhea among under-five children. Earlier research indicated that environmental sanitation, the type of drinking, and hygienic practice in households are the major determinants of under-five diarrhea, especially in rural areas of developing nations. To the best of our knowledge, there is no sufficient study in Ethiopia that determined the impact of environmental sanitation, drinking water, and other predisposing environmental factors among under-five children using large sample size data obtained from specific surveillance sites over a wider time period. Therefore, this study investigated the prevalence of diarrhea and its determinants among under-five children in the Kersa Demographic Health Survey in eastern Ethiopia.

According to the results obtained from the surveillance site, the prevalence of diarrhea among under-five children was 41.75%. This result is comparable with the previous report from North Uganda of 44.6% (17). However, the pooled prevalence of diarrhea in this finding is much higher than other findings from different areas of Ethiopia, including results from the EDHS with 16.1% (18), rural areas of the North Gondar zone with 22.1 % (19), Mecha district of west Gojam with 18.0% (11), Northwest Ethiopia with 25.3% (20), Sebeta town with 9.9%, Hadealeala with 26.1% (21), and Worabe town with 30.9 % (22). The prevalence of 2 week diarrhea from this surveillance site is also higher when compared to studies conducted in other countries like Rwanda with 26% (359), Malawi with 20% (14,872), Mbour Senegal with 26% (596), Tjo-Cameron with 23.8% (602), and India with 9% (23). However, the finding of this study is relatively low when compared to the finding of Enderta Woreda, Tigray region of Ethiopia of 54% (24).

The possible explanation for the discrepancy observed between studies is the difference in study duration. The data used in this study was relatively collected over a long period. However, most of the findings used for comparison in this study are results based on short-term studies, which cannot estimate the actual annual prevalence of diarrhea over time (25). Another possible explanation could be a difference in socioeconomic status, sample size, the accessibility of drinking water, and sanitation facilities (26). This indicates that more attention should be given to the provision of safe water and access to sanitation facilities in this area.

Mother-to-child media exposure is found to be significantly associated with a prevalence of 2 week diarrhea among under-five children. The odds of diarrhea among under-five children are 22% [AOR: 0.78 CI: (0.61, 0.98)] less likely among children whose mothers had media exposure when compared to their counterparts. Similarly, an independent study conducted showed that diarrhea is prevalent among children whose mothers cannot read and write (27). Another independent study conducted in North West Ethiopia and Nigeria also found that a mother's level of education had a strong significant association with the occurrence of under-five diarrhea (28). The possible justification may be because the hygienic practices of a mother during childcare have a significant impact on childhood diarrhea (27, 29).

Regarding the type of source of drinking water, our study showed that the likelihood of diarrhea was 50% higher among respondents who rely on unimproved drinking water sources. This finding is in line with the study conducted in north Ethiopia (30), south Ethiopia (31), west Ethiopia (32), northwest Ethiopia (29), and northwest Tigray (33). This could be attributed to the fact that unprotected water sources have a higher likelihood of being fecal-contaminated than unprotected water sources.

### Strengths and limitations of the study

Because of the cross-sectional nature of the data, it was challenging to draw any inferences about causation. Due to the secondary source of data, some important variables were lost. Despite these limitations, the findings of the present study were based on data that were representative. As a result, these findings offer data that programs and policymakers may utilize to cut down on diarrhea among under-five children in Eastern Ethiopia.

## Conclusion

The result of this study indicated that there is a high prevalence of under-five diarrhea in Kersa HDSS from 2016 to 2021. The main predictors of under-five diarrhea were a lack of latrines, an unimproved drinking water source, and a long distance from drinking water. This study area should therefore prioritize improving access to safe drinking water and sanitation. Moreover, appropriate health education focusing on the above-mentioned risk factors for diarrhea should be provided to the mothers to tackle diarrhea.

## Data availability statement

The original contributions presented in the study are included in the article/supplementary materials, further inquiries can be directed to the corresponding author.

## Ethics statement

The studies involving human participants were reviewed and approved by Haramaya University, College of Health and Medical Sciences, Institutional Health Research Ethical Review Committee. Written informed consent to participate in this study was provided by the participants' legal guardian/next of kin.

## Author contributions

All authors contributed to data analysis, drafting or revising the article, have agreed on the journal to which the article will be submitted, gave final approval of the version to be published, and agreed to be accountable for all aspects of the work.

## Conflict of interest

The authors declare that the research was conducted in the absence of any commercial or financial relationships that could be construed as a potential conflict of interest.

## Publisher's note

All claims expressed in this article are solely those of the authors and do not necessarily represent those of their affiliated organizations, or those of the publisher, the editors and the reviewers. Any product that may be evaluated in this article, or claim that may be made by its manufacturer, is not guaranteed or endorsed by the publisher.
